# The Impact of Hurricane Matthew on School Attendance: An Analysis from Rural Haiti

**DOI:** 10.3390/ijerph16010055

**Published:** 2018-12-26

**Authors:** Amanda C. Cook, Donovan Beachy

**Affiliations:** Department of Economics, Bowling Green State University; Bowling Green, OH 43403, USA; drbeach@bgsu.edu

**Keywords:** Natural disaster, human capital, education and economic development, education and inequality, school attendance, hurricane, probit

## Abstract

This study identifies the impact of Hurricane Matthew on school attendance in an agricultural community in rural Haiti. We conducted a survey of parents whose children attended a rural school prior to Hurricane Matthew to determine the mechanism by which hurricanes impact school attendance. We determined the marginal effect of family size and school enrollment using a probit model. Parents identified two primary causes for their children leaving school: a loss of income—through crop damage and livestock deaths—and requiring the children’s labor on the family farm. In our sample 96 children, 46% of the children enrolled in school, stopped attending because of the hurricane. No parent reported that their child(ren) left school because of illness or injury. Families with more children in school before the storm were 5% (*p* < 0.001) more likely to have a child remain in school. Families with some children not attending school before the hurricane were 7.6% (*p* < 0.001) more likely to leave school after the storm. The survey and probit model both suggest that an income constraint caused children to leave school. There is limited empirical evidence that students leave school to provide labor on family farms, and no evidence they leave school because of illness or injury.

## 1. Introduction

The intensity and frequency of hurricanes in the Caribbean has increased in the past few decades. While natural disasters have been studied for their impact on property damage, the environment, and economic well-being [1], it is challenging to quantify their effects on ‘softer’ metrics such as changes in health or reduced school attendance. In part, it is difficult to disentangle the social effects of natural disasters, as the disaster itself may induce migration. In developed countries, re-building efforts take time, so individuals may relocate to a nearby town, county, or state after a disaster. In developing countries, the likelihood of substantial government intervention is reduced. Depending on the country’s economic fundamentals, individuals may not receive much, or any, government assistance. Indeed, in the absence of the robust governmental responses to natural disasters seen in developed countries, developing countries are left with limited choices for disaster response. With limited governmental assistance, low socioeconomic-status individuals are more likely to remain in the same community after a storm, with fewer options for recovery. We identified the impact of Hurricane Matthew, which hit Haiti on 4 October 2016, on school attendance in an agricultural community, Dessab. We capitalized on a novel data set—a survey of parents—to determine why their children stopped attending school. Our paper provides insight into the mechanism by which hurricanes impact school attendance and quantifies how many students left school as the result of the storm. 

Dessab is a rural, mountain community about six miles north east of Arcahaie. There is a dirt road which connects the village to the highway that runs along the coast, but the road frequently washes out when it rains, making it unusable for vehicles. As such, the community is geographically isolated. Additionally, the community is comprised of small farms. A non-local land owner rents the farmland to the local families who pay their rent with crops at harvest time. Residents build homes on small bits of non-arable land adjacent to the land they cultivate. Usually, the residents own the small piece of land on which their home is built, though this is not legally documented. The community respects historical squatters’ rights, and homes are passed down within families. The relative geographic isolation of the community means that individuals do not have a formal relationship with financial institutions. As such, savings and wealth acquisition take the form of acquiring livestock. In this mountainous community, it is not uncommon for animals to get swept away in intense storms. As such, this means negative wealth shocks are frequently correlated. Hurricanes destroy crops, diminishing current income, and injure or kill livestock, eliminating past savings. The median income in this community is less than $2 a day. These institutional details are important because they help to illustrate that there is very limited potential for geographic mobility. 

Like the majority of schools in Haiti, the Institution Mixte du Progres in Dessab is funded through tuition payments. After the hurricane, with many families experiencing both an immediate income shock (with the loss of livestock) and an extended shock (in the form of extensive crop damage), families may be unable to continue to pay for their child(ren) to continue at school. 

Toya and Skidmore [2] highlight a key issue when considering the impact of a hurricane: the most vulnerable communities include those which have low levels of education. However, the hurricane itself causes a reduction in school attendance, creating a cyclical pattern where those with low levels of education are the hardest hit by a natural disaster and one effect is to reduce the probability children will continue in school, causing the community to be stuck in a negative feedback loop. As Pichler and Striessnig [3] show, this negative cycle is prevalent in Haiti, which ranks highest among Caribbean countries in social vulnerability to hurricanes.

Furthermore, as Skoufias [4] posits, nondeveloped countries are also the slowest to fully recover from natural disasters, socially, economically, and infrastructurally, thereby furthering the effects of the aforementioned negative feedback loop. Kahn [5] found that economic development acts as insurance against disasters due to a higher level of state expenditure on disaster response measures; richer countries are not only able to respond to a disaster more effectively, they are also preemptively prepared. This early expenditure ensures long-term economic and social well-being as communities are able to more quickly return to their pre-disaster state, thereby preventing the stagnation in human capital development. Given these factors, Albala-Bertrand’s [6] findings that weaker economies and political bases contribute to larger effects of disasters are no surprise. Considering the aggregated hypotheses of these authors, one might expect the results of this study to provide an upper bound on the impact of a storm on school enrollment. The community in Dessab, like much of Haiti, is highly susceptible to negative impacts from natural disasters due to its low economic development and weak government.

We find that nearly half the students at the Institution Mixte du Progres left school after the storm. Students were 5% more likely to remain in school if they had a sibling enrolled in school. Students were 7.6% more likely to leave school if they had a sibling that was not enrolled in school before the storm. As tuition is costly, we view this as evidence of wealth protecting students from the impact of natural disasters. This empirical work aligns with our survey result.

## 2. Materials and Methods

Parents of children who attended the Institution Mixte du Progres in October 2016 were contacted to complete a survey. Approximately 55 families were surveyed. Surveys were completed by a parent, both in writing or orally depending on the literacy of the parents. A copy of the survey, translated into English, can be found in Appendix A (All subjects gave their informed consent for inclusion before they participated in the study).

Additionally, we employed a probit model to determine what factors predicted a child’s continuation in or withdrawal from school.

(1) $$KidLeft_{i}=\beta_{0}+\beta_{1}\times NumberChildrenInSchool_{i}+\beta_{2}\times NumberChildrenNotInSchool_{i}+\epsilon_{i}$$

Errors are clustered at the family level. *KidLeft* is a dummy variable equal to 1 if the child left school. *NumberChildrenInSchool* is a variable that counts the number of siblings in a family enrolled in school. *NumberChildrenNotInSchool* is a variable that counts the number of siblings in a family that were not enrolled prior to Hurricane Matthew. 

## 3. Results and Discussion

**Figure 1:**
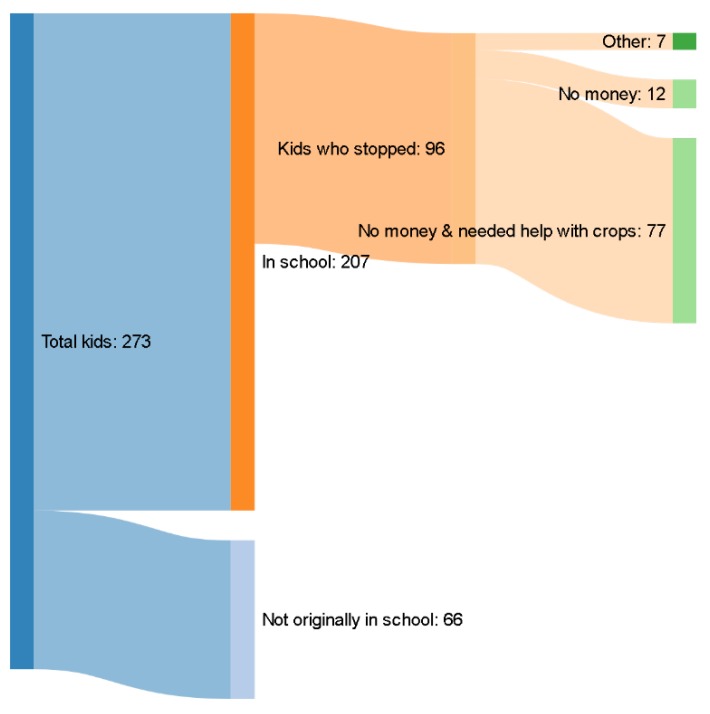
Survey results from parents of children at the institution Mixte du Progres in Dessab in 2016.

As one can see in Figure 1, at the start of the school year there were 273 children in the community, of whom 207 were enrolled in school. After the hurricane, 96 withdrew from school. Of the children who withdrew, 80% of parents attributed their withdrawal to both an income constraint and the family needing help on the farm to replant crops. Parents of an additional 12 children withdrew because of finical constraints only. Fewer than 10 percent of the children were withdrawn for other reasons, none related to illness or injury. 

Next, we wanted to determine if we could predict the likelihood of a student leaving school based on their family characteristics. Using Equation (1), we estimated the probit model and then calculated the marginal effect of each variable on a child leaving school. Table 1 contains the results. 

The marginal effect of the *Number of Siblings at School* is –0.0482 (*p* < 0.001). This means that for each additional child in a family in school, the probability a child leaves school *decreases* by 4.8 percentage points. The child is *more* likely to stay in school. Similarly, the marginal effect of the *Number of Siblings Not at School* is 0.0758. This means that for each additional child in a family, the probability one leaves school *increases* by 7.6 percentage points (*p* < 0.001). Taking these two results together provides evidence of the impact of a family’s wealth on a child’s continued enrollment. Having multiple children in school is costly, as school is tuition based. Those families which could afford to have more children in school before the storm were ones with additional financial resources. Additionally, these results are suggestive that the constraint is not related to child labor. If children were leaving school solely to provide labor on their families’ farms, we would anticipate the marginal effect of *Number of Siblings Not at School* to be negative. Families who had children that were not enrolled in school could employ those non-enrollees to replant crops. Thus, having a sibling out of school, who could provide labor, should buffer the enrolled child from the impact of the storm and help that child remain in school. However, we see the opposite sign: an additional sibling who is not enrolled in school increases the likelihood of a previously-enrolled child leaving school. 

## 4. Conclusions

After Hurricane Matthew, nearly half the children at the Institution Mixte du Progres left school. Both survey results and empirical analysis suggest that this was largely due to an income constraint. Survey results indicate that the children were also needed to work on family farms, though the empirical results suggest that this motivation was secondary. Families with more children in school before the storm were approximately 5 percentage points less likely to have a child leave school. Families with more children who were not enrolled in school before the storm were approximately 7.5 percentage points more likely to have a child leave school. 

This paper contributes to the literature by identifying the motivation for withdrawing from school. It is largely financial. It is significant to observe that for this village, neither illness nor injury was listed as a cause for having a child leave school. Further, it demonstrates heterogeneity in a family’s response based on wealth.

As we think about climate change and more intense or more frequent storms, there is a disproportionate impact on very vulnerable populations. While the impact of natural disasters is well studied in developed countries, in less-developed countries, less is known about short- and long-term effects of disasters. While it is easy to focus on first order effects—total damages to property, lives lost, etc.—the second order effects, such as interrupted schooling, are both significant and have long-term consequences. This paper contributes to our understanding of how vulnerable populations weather the storm.

It also is important to consider how natural disasters create a cycle of poverty. Literature identifies low educational attainment as a factor for countries having a difficult time navigating the effects of a disaster. However, that very disaster causes reductions in school attendance. Disasters have a significant impact on some communities because of low levels of education, then the disaster itself causes students to leave school, which intensifies the problem. Breaking this cycle will be an important key in reconciling the perpetual poverty in which some countries find themselves. As we consider the impact of global climate change, we should consider not only the direct impact these increased natural disasters have now, but also the effect they will have for future generations.

## Figures and Tables

**Table 1:** The Marginal Effects of an Additional Sibling Enrolled or not Enrolled in School Prior to the Hurricane on the Probability a Student Continued Enrollment.

Title 1	Marginal Effects
Number of Siblings at School	−0.0482 *** (−3.87)
Number of Siblings Not at School	0.0758 *** (3.81)

t statistics in parentheses: * *p* < 0.05, ** *p* < 0.01, *** *p* < 0.001.

## Appendix A

English Translation of the Survey given to Parents of Children the Institution Mixte du Progres in Dessab in 2016:

1. How many children do you have?
2. How many children went to the Institute de Progress in Dessab last school year (2016–2017)?
3. Did any of your children stop going to school after the hurricane in October? Yes  No
4. How many stopped going?
5. Why did they stop?
  (a) No money for school;
  (b) The child was sick or injured;
  (c) Needed help with planting crops;
  (d) Other.
